# Single-port VATS combined with non-indwelling drain in ERAS: a retrospective study

**DOI:** 10.1186/s13019-021-01657-x

**Published:** 2021-09-26

**Authors:** Jiantian Yang, Wencong Huang, Peijian Li, Huizhen Hu, Yongsheng Li, Wei Wei

**Affiliations:** 1 Department of Cardiothoracic Surgery, Huizhou Municipal Central Hospital, 41 Eling North Road, Huizhou, 516001 China; 2 Department of Pathology, Huizhou No. 1 Maternal and Child Care Service Center, Huizhou, 516007 China

**Keywords:** Enhanced recovery after surgery, Single-port thoracoscopy, Non- indwelling drain

## Abstract

**Background:**

We investigated single-port video-assisted thoracoscopic surgery (VATS) combined with a postoperative non-indwelling drain in enhanced recovery after surgery (ERAS).

**Methods:**

The clinical data of 127 patients who underwent double- and single-port VATS from January 2018 to December 2019 were analyzed retrospectively. The groups constituted 71 cases undergoing double-port and 56 cases undergoing single-port VATS (30 cases in the indwelling drain group and 26 cases in the non-indwelling drain group). The incidence of postoperative complications, pain scores, and postoperative hospital stay were compared between the two groups.

**Results:**

Compared with the double-port group, the single-port group had shorter postoperative hospital stays and lower pain scores on the first and third postoperative days (P < 0.05). Pain scores on the first and third days were lower in the single-port non-indwelling drain group than in the single-port indwelling drain group (P < 0.05), and the postoperative hospitalization time was significantly shorter in the single-port group (P < 0.05). However, there was no significant difference between the two groups for operation time, incidence of complications, and pain scores 1 month after operation (P > 0.05).

**Conclusions:**

The combination of single-port VATS with a non-indwelling drain can relieve postoperative pain, help patients recover quickly, and is in accordance with ERAS.

## Background

Enhanced recovery after surgery (ERAS) is a new medical and nursing concept involving a series of perioperative evidence-based optimized treatments to reduce patients’ physiological and psychological stress when undergoing surgery, and to achieve accelerated rehabilitation [1, 2]. The ERAS concepts are becoming more widely used in clinical practice in many countries in Europe, with good results; however, currently, ERAS is being used relatively infrequently in thoracic surgery [3, 4]. Traditional VATS entry into the chest cavity has decreased from four to two ports and to as low a single port [5–8]. With the change from the traditional postoperative indwelling thoracic drain to the current drain-free surgery, patients’ pain is further alleviated, and the impact on sensory and motor function is minimized [9–11], which is fully in line with the ERAS concepts. This study retrospectively analyzed 127 cases of double- and single-port thoracoscopic surgery, and discussed the application of single-port thoracoscopic surgery combined with a non-indwelling drain in ERAS.

## Methods

### Patients

We reviewed the records of all patients who underwent double- and single-port thoracoscopic surgery from January 2018 to December 2019 at the Huizhou Central People's Hospital in Guangdong, China. The inclusion criteria were: 1. age from 18 to 70 years; 2. peripheral benign pulmonary nodules or metastases, bullae, or mediastinal masses (lung wedge resection or mass resection); 3. double- or single-port VATS; and 4. no serious heart, liver, kidney, or other underlying diseases affecting postoperative recovery or prolonging hospital stay. There were 71 cases in the double-port group and 56 cases in the single-port group. The single-port group was divided into 30 cases in the indwelling drain group and 26 cases in the non-indwelling drain group, classified on the basis of whether the chest drain was retained after surgery.

### Surgical technique

All patients underwent general anesthesia with bronchial intubation and lung ventilation on the healthy side. The surgeon and first assistant stood on the same side of the patient, the patient's ventral side. An incision was made in the 4th or 5th intercostal space on the affected side. In the double-port group, we added a port for the endoscope in the 6th or 7th intercostal space. The incision measured approximately 3–4 cm along the intercostal space between anterior and axillary lines. We placed wound edge protector inside the incision, and used a 30° thoracoscope for thoracic exploration. The suction device was combined with an electrocoagulation hook and ultrasonic knife for surgical operation. After the surgery, a chest tube (16 F stomach tube) was inserted into the observation port in the double-port group or into the incision in the single-port indwelling drain group. The incision was sutured closed layer by layer. In the single-port non-indwelling drain group, the drain was not fixed, and the anesthesiologist was instructed to fully inflate the lungs, then the chest tube was removed under negative pressure, and the patient entered the anesthesia resuscitation room. After patients regained consciousness, the tracheal intubation tube was removed, patients returned to the general ward for electrocardiographic monitoring, routine fluid replacement, and analgesia (flurbiprofen axetil 50 mg, intravenous injection, once every 12 h). Bedside chest X-ray examination was performed the day after operation.

### Variables

Operation time, postoperative hospital stay, postoperative pain score (first and third postoperative days and the first month after operation), postoperative complications (pulmonary infection, active thoracic hemorrhage, atelectasis, pneumothorax (lung compression > 30%), incision infection) were recorded in the three groups. A 10-point visual analogue scale (VAS) was used to evaluate postoperative pain. The score ranged from in 0–10, where 0 indicated no pain; 1–3 indicated mild endurable pain; 4–6 indicated moderate pain that was endurable but affected sleep quality; 7–9 indicated progressive unendurable pain; and 10 indicated severe pain. Higher scores indicated more severe pain.

### Statistical analysis

All data were analyzed using SPSS 22.0 statistical software (IBM Corp., Armonk, NY). Measurement data (mean age, operative time, postoperative hospital stay, and VAS scores) were expressed as mean ± SD. The independent samples t-test was used to compare differences between groups. Numerical data (sex, smoking history, concurrent disease, postoperative complications) were expressed as n (%), and values were compared between the groups using Fisher’s exact test or the chi-square test. P < 0.05 indicated that the difference was statistically significant.

## Results

The results of the comparison of the pre- and postoperative data between the double-port group and the single-port group: Table 1 shows the distributions for sex, age, history of smoking, and type of operation, and no significant differences were found (P > 0.05). In Table 2, there was also no significant difference between these two groups regarding the operation time and postoperative complications (P > 0.05). Compared with the double-port group, the postoperative hospital stay in the single-port group was slightly shorter, and the difference was statistically significant (P < 0.05). The pain scores in the single-port group decreased significantly on the first and third postoperative days (P < 0.05), and there was no significant difference between the two groups 1 month postoperatively (P > 0.05). Pulmonary infection occurred in six cases, and incisional wound infection occurred in two cases postoperatively, in the double-port group.

**Table 1:** Demographic and clinical characteristics of double-port and single-port

Characteristics	Double-port	Single-port	P
Sex (male/female)	39/32	33/23	0.720
Age (years,mean ± SD)	43.39 ± 13.07	41.20 ± 11.75	0.327
History of smoking (yes/no)	21/50	17/39	0.924
Type of operation			0.701
Lung wedge resection n (%)	50 (70.42)	37 (66.07)	
Mass resection n (%)	21 (29.58)	19 (33.93)	

P < 0.05 is considered statistically significant

**Table 2:** Intraoperative and postoperative parameters

Parameters	Double-port	Single-port	P
Operation time (min, mean ± SD)	54.86 ± 13.63	57.86 ± 13.34	0.216
Postoperative complications n (%)	8 (11.26)	5 (8.93)	0.666
Postoperative hospital stay (day, mean ± SD)	5.62 ± 1.60	4.57 ± 2.13	0.003
Pain score on the first day after operation	4.77 ± 1.33	4.05 ± 1.51	0.006
Pain score on the third day after operation	4.38 ± 1.29	3.64 ± 1.33	0.002
Pain score on first month after operation	1.23 ± 0.93	1.21 ± 0.95	0.950

P < 0.05 is considered statistically significant

The results of the comparison of the pre- and postoperative data between the indwelling and non-indwelling drain groups in the single-port group: Table 3 shows the distributions for sex, age, history of smoking, and type of operation, and no statistically significant differences were found (P > 0.05). In Table 4, there was also no significant difference in operative time and postoperative complications between the indwelling drain group and non-indwelling drain group (P > 0.05). Comparing the indwelling drain group with the non-indwelling drain group, postoperative hospital stay in the non-indwelling drain group was obviously shorter (P < 0.05). On the first and third postoperative days, pain scores in the non-indwelling drain group decreased significantly (P < 0.05), but there was no significant difference in the pain scores 1 month postoperatively (P > 0.05). Two patients developed pulmonary infection, and one patient developed incisional infection in the indwelling drain group. All three patients were treated with antibiotics, and more frequent wound disinfection and dressing changes. Complications occurred in two patients in the non-indwelling drain group. One of these patients developed pneumothorax (with 30% lung compression) on the day of surgery. X-ray examination was repeated on the third day after conservative treatment with oxygen therapy, and images showed that the severity of the pneumothorax had decreased to 15% lung compression. The second patient developed pulmonary infection after surgery, and was discharged after 6 days of antibiotic therapy. Both patients recovered well and did not require repeat drainage.

**Table 3:** Demographic and clinical characteristics of indwelling drain and non-indwelling drain

Characteristics	Indwelling drain	Non-indwelling drain	P
Sex (male/female)	17/13	14/12	0.832
Age (years, mean ± SD)	42.70 ± 11.98	39.46 ± 11.46	0.308
History of smoking (yes/no)	11/19	5/21	0.236
Type of operation			0.384
Lung wedge resection n (%)	19 (63.33)	20 (76.92)	
Mass resection n (%)	11 (36.67)	6 (23.08)	

P < 0.05 is considered statistically significant

**Table 4:** Intraoperative and postoperative parameters

Parameters	Indwelling drain	Non-indwelling drain	P
Operation time (min, mean ± SD)	57.67 ± 12.91	58.08 ± 14.08	0.910
Postoperative complications n(%)	3 (10)	2 (7.69)	1.000
Postoperative hospital stay (day, mean ± SD)	5.80 ± 1.63	3.15 ± 1.74	< 0.001
Pain score on the first day after operation	4.50 ± 1.55	3.54 ± 1.30	< 0.001
Pain score on the third day after operation	4.37 ± 1.13	2.81 ± 1.02	0.012
Pain score on one month after operation	1.37 ± 0.93	1.04 ± 0.72	0.143

P < 0.05 is considered statistically significant

## Discussion

ERAS is a multi-disciplinary approach involving anesthesia, nursing care, and surgery. The core concepts involve optimizing patients’ perioperative treatment and nursing measures, reducing complications and patients’ stress response, and accelerating rehabilitation. Thoracic surgery involves recent changes from traditional thoracoscopic surgery to single-port thoracoscopy and the introduction of Da Vinci robotic surgery (Intuitive Surgical, Sunnyvale, CA). Anesthesia methods have also changed to attempting anesthesia without endotracheal intubation, and postoperative management now involves analgesia without a catheter or a drain [12–15]. The results of this study further support that single-port thoracoscopic surgery combined with a postoperative non-indwelling drain can significantly reduce surgical trauma and postoperative pain. Postoperative pain relief is very important in promoting the rapid recovery of surgical patients.

The possible causes of pain after thoracic surgery arise mainly from: 1. pain from the incision itself and compression of the intercostal nerve during and after operation; 2. an over-long or kinked chest tube after operation and stimulating the diaphragm or the parietal pleura; and 3. psychological factors; many patients fear the chest drain and incision after operation as well as the pain caused by managing the chest tube, which causes additional psychological stress. The results of this study show that performing single-port thoracoscopic surgery with no postoperative indwelling drain can decrease early postoperative pain. Postoperative pain, specifically, does not exacerbate the disease, but pain can lead to poor postoperative rehabilitation and may lead to further exacerbation of the disease. Clinically, patients in both the double-and single-port drain group must carry a drainage bottle when they get out of bed, and pain is aggravated when they walk, so most do not want to get out of bed on the first postoperative day. Additionally, postoperative pain affects coughing and sputum drainage, which cannot be effectively discharged. In elderly patients, this effect of pain greatly increases the risk of pulmonary infection, atelectasis, and other complications [16–20].

Single-port VATS without a postoperative indwelling drain eliminates having to remove the drain. **Since** there is no need to maintain a chest tube as long as there are no obvious abnormalities on postoperative chest X-rays, the discharge standard can be reached, postoperative hospitalization time can be shortened, and the economic burden on patients and their families can be reduced. Furthermore, not having a drain minimizes psychological stress caused by long hospital stays and increases the possibility of progressing to day surgery for these procedures, in the future.

The results of this study showed that there was no statistically significant difference in postoperative bleeding, infection, and other complications between patients in the double-port group, single-port indwelling drain group, and the single-port non-indwelling drain group. It is reasonable and feasible not to place a drain after operation, and doing so is more conducive to patients’ rehabilitation. However, single-port thoracoscopic pulmonary wedge resection and mediastinal resection without retention of a chest tube still must follow certain principles. These principles are minimizing intraoperative bleeding and exudation, and avoiding senile emphysema and pulmonary infection. There must also be minimal possibility of postoperative lung leakage and pleural effusion. In the next step, we will expand the cases in each group to further study the adverse events of single-port VATS without a postoperative indwelling drain. However, whether single-port VATS combined with a non-indwelling drain can be extended to lobectomy or sleeve resection and other more involved procedures remains to be further studied [21, 22].

## Conclusions

Single-port thoracoscopic surgery combined with non-indwelling drain after surgery can better reduce postoperative pain, shorten postoperative hospital stay, and does not increase the risk of postoperative complications, which is conducive to enhanced recovery after surgery.

## Data Availability

The datasets used and/or analysed during the current study are available from the corresponding author on reasonable request.

## Abbreviations

VATS: Video-assisted thoracic surgery
ERAS: Enhanced recovery after surgery
VAS: Visual analogue scale
